# Intrinsic DMI-free skyrmion formation and robust dynamic behaviors in magnetic hemispherical shells

**DOI:** 10.1038/s41598-021-81624-7

**Published:** 2021-02-16

**Authors:** Jaehak Yang, Claas Abert, Dieter Suess, Sang-Koog Kim

**Affiliations:** 1 National Creative Research Initiative Center for Spin Dynamics and Spin-Wave Devices, Nanospinics Laboratory, Research Institute of Advanced Materials, Department of Materials Science and Engineering, Seoul National University, Seoul, 151-744 South Korea; 2 Faculty of Physics, University of Vienna, Vienna, Austria; 3 University of Vienna Research Platform MMM Mathematics - Magnetism - Materials, University of Vienna, Vienna, Austria

**Keywords:** Magnetic properties and materials, Topological defects

## Abstract

We performed finite-element micromagnetic simulations to examine the formation of skyrmions without intrinsic Dzyaloshinskii–Moriya interaction (DMI) in magnetic hemispherical shells. We found that curvature-induced DM-like interaction allows for further stabilization of skyrmions without the DMI in curved-geometry hemispherical shells for a specific range of uniaxial perpendicular magnetic anisotropy (PMA) constant *K*_*u*_. The larger the curvature of the shell, the higher the *K*_*u*_ value required for the formation of the skyrmions. With well-stabilized skyrmions, we also found in-plane gyration modes and azimuthal spin-wave modes as well as an out-of-plane breathing mode, similarly to previously found modes for planar geometries. Furthermore, additional higher-frequency hybrid modes were observed due to coupling between the gyration and azimuthal modes. This work provides further physical insight into the static and dynamic properties of intrinsic DMI-free skyrmions formed in curved-geometry systems.

## Introduction

Chiral spin textures such as chiral domain walls^1^, vortices^2^, and skyrmions^3,4^ are considered to be promising building blocks for prospective memory and logic devices in the field of spintronics^5–10^. In particular, the magnetic skyrmion has been intensively studied owing not only to its novel fundamental static and dynamic properties^11–15^ but also to its technological applications to information-storage and -processing devices^16,17^. The characteristic features of skyrmions include nano-scale size, topological stability, and the ultra-low current density required for its motion, making it a promising information carrier for real-device applications^18–20^.

In order to form and stabilize skyrmions in thin layered films for practical implementations in real information storage and processing devices, intrinsic DMI^21,22^ favoring non-collinear spin configurations, together with strong spin–orbit interaction at the interfaces of the layered films, is necessary. Since the strength of DMI is limited to less than ~ 2 mJ/m^2^ in two-dimensional (2D) planar thin films with perpendicular magnetic anisotropy (PMA)^23,24^, exploration of alternative systems wherein skyrmions are further stabilized is challenging. To that end, recent theoretical and experimental studies have revealed that three-dimensional (3D) curved geometries and torsional magnets can allow for robust effective magnetic interactions of curvature-induced effective magnetic anisotropy and DM-like interaction^25–33^. For example, Carvalho-Santos et al.^33^ reported that the curved geometry can enforce the effective DMI, while it makes a certain reduction in the effective magnetic anisotropy, thereby leading to a further stability of skyrmion formation. Thus, the combination of these two energy terms will make possible significant modifications of the stability and dynamical properties of skyrmions in geometrically curved dots, even without intrinsic DMI.

Here, we demonstrate that, even in the absence of both intrinsic DMI and applied magnetic fields, curved-geometric confinements are another efficient means of stabilizing the topological texture of a skyrmion with the help of uniaxial magnetic anisotropy. For example, we found that sufficiently stable skyrmions can be formed in a certain range of uniaxial PMA constant in specially designed hemispherical shells. We also investigated robust dynamic fundamental and hybrid modes of single skyrmions in hemispherical shells. Our findings further the understanding of skyrmion stability in curved geometries without DMI, and additionally, offer an efficient means of controlling robust skyrmion dynamics for real-device applications.

## Results

### Model geometry

**Figure 1:**
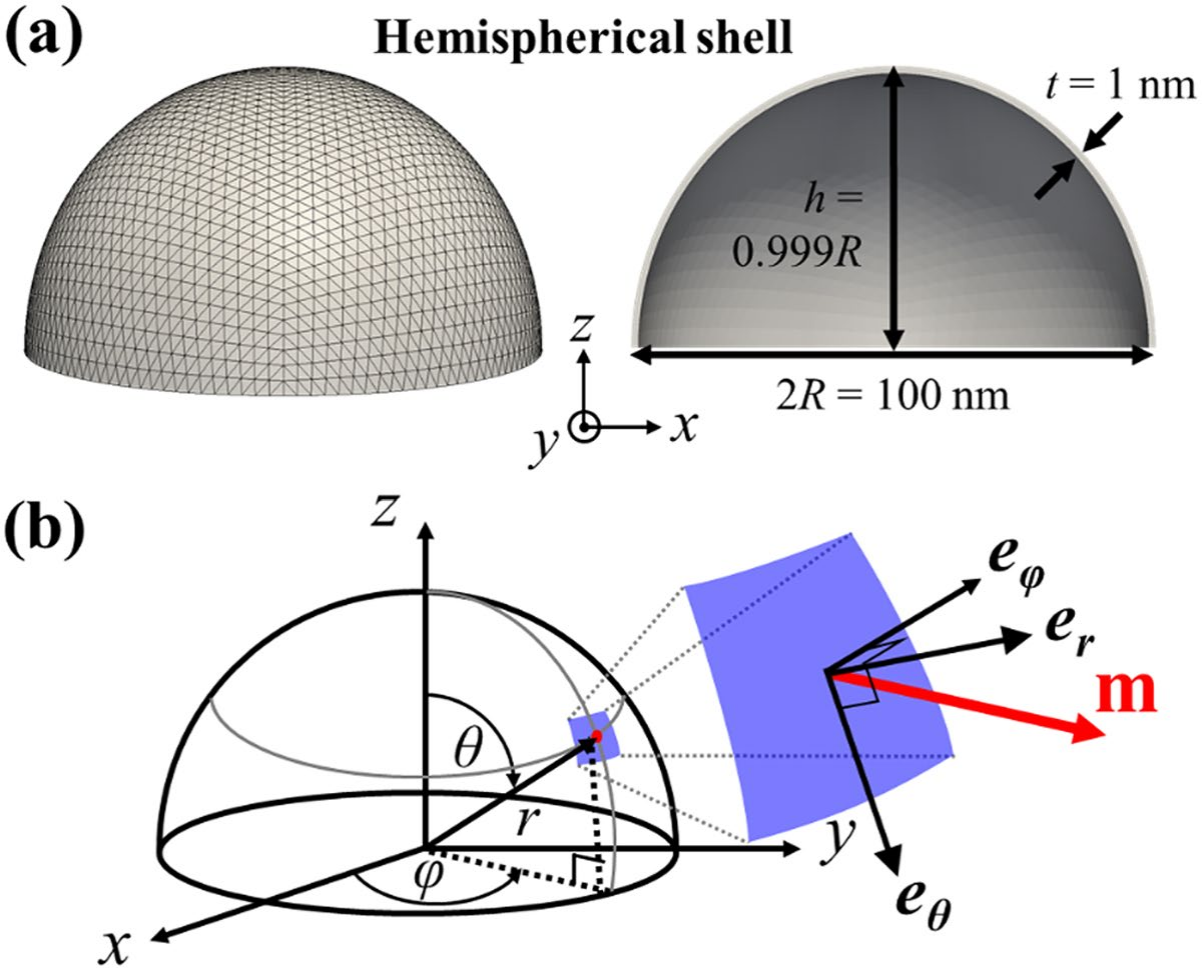
Finite-element model geometry and coordinate systems. (**a**) Modeling of finite-element spherical shells of diameter 2*R* = 100 nm, height *h* = 0.999*R*, and shell thickness *t* = 1 nm. (**b**) Representation of spherical coordinates and local spherical reference frame of local surface (blue-color surface) for local magnetization *m*. Three different unit vectors, *e*_*r*_, *e*_*θ*_, and *e*_*φ*_ on the local surface, are represented by spherical coordinates.

The model system employed was half-spherical shells of *t* = 1 nm thickness for different outer diameters in the range of 2*R* = 25–100 nm. Since thin magnetic films can have different interfacial magnetic anisotropy depending on their thickness^34^, we also varied the uniaxial magnetic anisotropy constant *K*_*u*_, even for a single thickness of *t* = 1 nm, in the present micromagnetic simulations. Figure 1a shows an example of a hemispherical shell of 2*R* = 100 nm. The 3D volume of the model was discretized into tetrahedrons with an average node distance of 1 nm. We also discretized the curved surfaces into triangles of roughly equal area using Hierarchical Triangular Mesh^35^, thereby preventing numerical errors incurred by irregularities. For all hemispherical shells used in the simulations, the height (*h*), the distance to the outer top surface along the *z*-axis from the base (see Fig. 1a) was set to 99.9% of the given radii *R* in order to initially place the core of a single skyrmion at the center. This geometry does not change the overall static and dynamic characteristics of skyrmions.

For the sake of convenience in the analysis of simulation data obtained from the hemispherical shells, we converted the numerical data in the frame of Cartesian coordinates (*x*, *y*, *z*) to the corresponding data in the frame of spherical coordinates (*e*_*r*_, *e*_*θ*_, *e*_*φ*_) on every local surface (see Fig. 1b). The unit vector of local magnetizations is expressed as $(m_{r} ,m_{\theta } ,m_{\varphi } ) = ({\mathbf{m}} \cdot {\mathbf{e}}_{{\mathbf{r}}} ,{\mathbf{m}} \cdot {\mathbf{e}}_{\theta } ,{\mathbf{m}} \cdot {\mathbf{e}}_{\varphi } )$, where *r* is the radial distance, *θ* is the polar angle, and *φ* is the azimuthal angle in the frame of local spherical reference, as shown in Fig. 1b. For better visualizations of numerical data in our analysis, the static and dynamic data for the 3D curved system (e.g., the magnetization profiles and the skyrmion numbers of single skyrmions in hemispherical shells) are presented in unrolled 2D planar views according to the transformation formula $X = \frac{\theta }{\sin \theta }x\,,\,\,\,Y = \frac{\theta }{\sin \theta }y\,,\,\,\,{\text{and}}\,\,\,R^{\prime} = \frac{\pi }{2}R$, where (*X*, *Y*) and *R'* are the in-plane position in the equivalent disk and its radius in the frame of Cartesian coordinates, respectively.

### Spin textures including skyrmion formation without intrinsic DMI in hemispherical shells

**Figure 2:**
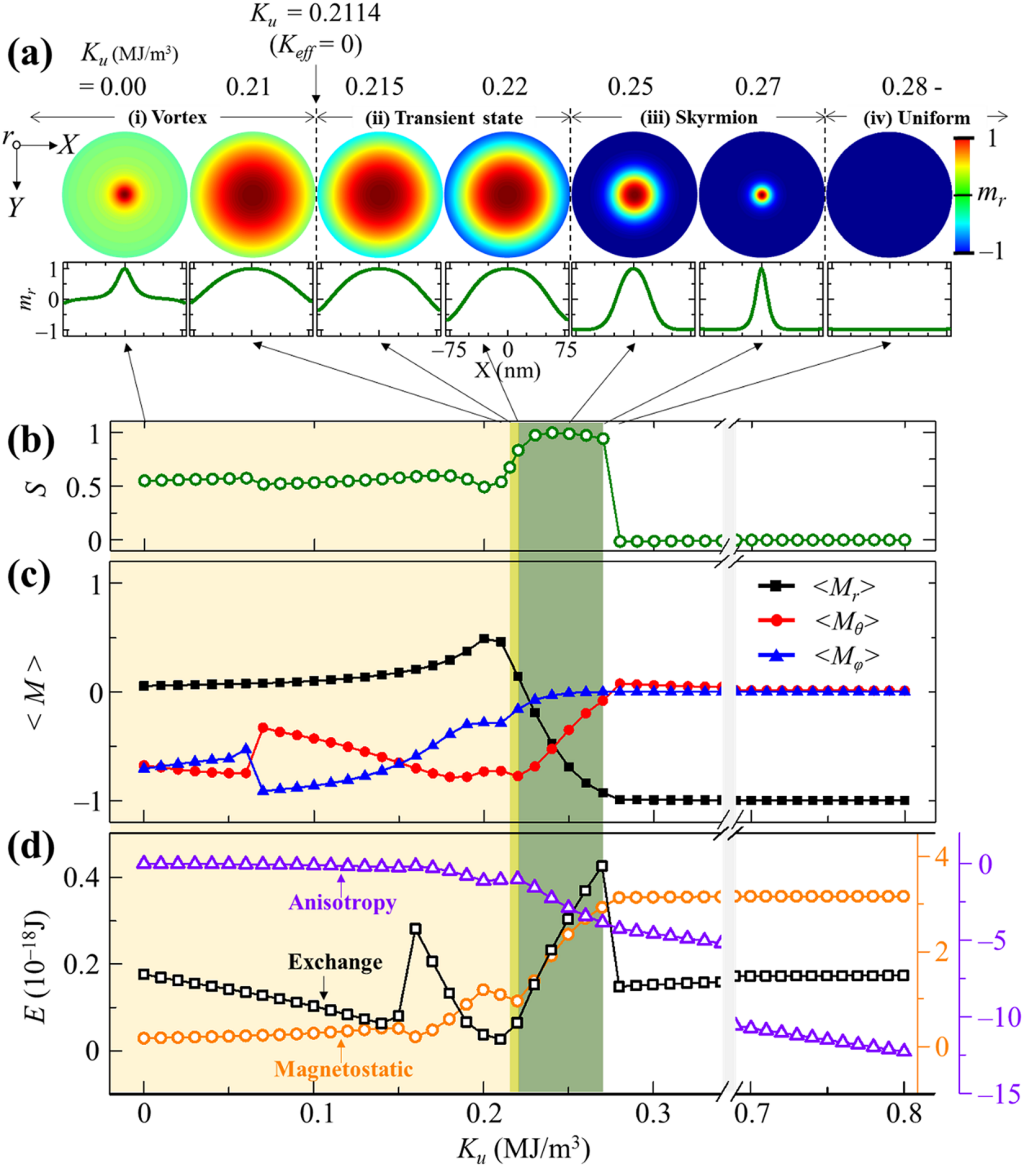
Variation of spin textures according to uniaxial perpendicular magnetic anisotropy (PMA) constant *K*_*u*_. (**a**) Unrolled-view images of spatial distributions of surface-normal magnetization component *m*_*r*_ (= *M*_*r*_/*M*_*s*_) of local magnetizations in corresponding ground states of hemispherical shell for 2*R* = 100 nm and different values of uniaxial anisotropy *K*_*u*_ with zero DMI constant. At the bottom, the corresponding *m*_*r*_ line profiles across the center of the half-shells are given. There are four different types of metastable state: vortex (*K*_*u*_ = 0 − 0.21 MJ/m^3^), transient state (*K*_*u*_ = 0.215 − 0.22), skyrmion (*K*_*u*_ = 0.23 − 0.27), and uniform magnetization state (*K*_*u*_ = 0.28 − 0.80). (**b**) Skyrmion number *S* and (**c**) spatially averaged magnetization components <*m*_*r*_>, <*m*_*θ*_>, and <*m*_*φ*_> and (**d**) exchange, magnetostatic, and anisotropy energy over entire shells as function of *K*_*u*_. The light yellow, orange, and green areas indicate the *K*_*u*_ section where the vortex, the transient state, and the skyrmion can be metastable, respectively.

Figure 2a shows ground (or at least meta-stable) states obtained from relaxation for 10 ns with *α* = 1 from the initially intended Néel-type skyrmion state for the noted different *K*_*u*_ values but zero DMI constant (*D*_*int*_ = 0) for a case of 2*R* = 100 nm. The corresponding line profiles of the normalized magnetization *m*_*r*_ (= *M*_*r*_/*M*_*s*_) across the shell center are displayed at the bottom of each *m*_*r*_ image. The local magnetization configurations could be classified into four different topological textures as a function of *K*_*u*_ employed in the simulations: (1) well-defined vortex (0 ≤ *K*_*u*_ ≤ 0.21 MJ/m^3^), (2) transient state from vortex to skyrmion (*K*_*u*_ = 0.215, and 0.22 MJ/m^3^), (3) well-defined skyrmion (0.23 ≤ *K*_*u*_ ≤ 0.27 MJ/m^3^), and (4) uniform magnetization state along surface normal (*K*_*u*_ ≥ 0.28 MJ/m^3^). These characteristic spin textures can be distinguished by the skyrmion number^36^, $S = \iint\limits_{A} {(1/4\pi )m \cdot (\partial_{X} m \times \partial_{Y} m)dXdY}$ with *A* the entire area in the unrolled disk geometry converted from the 3D hemispherical shape. Figure 2b shows the variation of *S* with *K*_*u*_. The topological textures of the vortex, transient state, skyrmion, and perpendicular magnetization states were classified by ~ 0.5, 0.5 < *S* < 1, ~ 1, and 0, respectively. Note that *S* for the skyrmion state is not exactly an integer as in infinite flat thin films, but rather is close to 1 due to its confined geometry. The local magnetizations tilted near the edge boundaries cause a non-integer skyrmion number (i.e., *S* = 0.94–0.99 for *K*_*u*_ = 0.23–0.27 MJ/m^3^) even for the stabilized skyrmion state. With increasing *K*_*u*_, *S* is continuously variable from the vortex to the skyrmion state, but is suddenly changed from ~ 1 (skyrmion) to 0 (uniform perpendicular magnetizations) (see Fig. 2b). Such clear transformation of the corresponding spin texture occur around very specific values of *K*_*u*_, such as 0.211, 0.224, and 0.274 MJ/m^3^. These characteristic *K*_*u*_ values can differently affect an effective magnetic anisotropy term in competition with curved-geometry-induced DM-like interaction for the formation of stable skyrmions even without DMI. Since the vortex state represents in-plane curling magnetization around the perpendicular magnetization at the core while the skyrmion state represents out-of-plane magnetizations in most areas, we can expect that the sign of the effective magnetic anisotropy constant *K*_*eff*_ affects the corresponding spin textures with the help of the curved-geometry-induced DM-like interaction. Roughly considering in-plane dipolar interaction on the local surfaces in such thin shells, we assume $K_{eff} = K_{u} - \frac{1}{2}\mu_{0} M_{s}^{2}$. Since *K*_*eff*_ < 0 and > 0 prefer in-plane and out-of-plane collinear spin ordering, respectively, we can readily expect that the value of *K*_*u*_ = 0.2114 MJ/m^3^ leads to *K*_*eff*_ = 0 with a given value of *M*_*s*_ = 580 kA/m for Co. The estimated value of *K*_*u*_ = 0.2114 MJ/m^3^ agrees well with the simulation result of spin-texture evolution according to *K*_*u*_. In the simulation result, for *K*_*u*_ < 0.2114 MJ/m^3^, the local magnetizations favor alignment in the surface plane (vortex states) except for the vortex core region, but for *K*_*u*_ > 0.2114 MJ/m^3^, they start to prefer alignment in the local surface normal (transient state, skyrmion, and perpendicular magnetization states).

For further details on the classified spin textures, we also plotted the three different magnetization components averaged over the entire area: radial <*m*_*r*_>, polar <*m*_*θ*_>, and azimuthal <*m*_*φ*_>, as shown in Fig. 2c, along with the exchange, magnetostatic, and magnetic anisotropy energy terms as a function of *K*_*u*_, as shown in Fig. 2d. The main features are described in detail below. In the vortex state (0 ≤ *K*_*u*_ ≤ 0.21 MJ/m^3^), the magnetization configuration consists of out-of-plane magnetizations at the core and in-plane curling magnetizations around the core. As *K*_*u*_ increases to 0.21 MJ/m^3^, the size of the vortex core increases, because the higher the *K*_*u*_, the more favorable are the out-of-plane magnetizations. The vortex state is also modified by the magnitude of *K*_*u*_. In two regions of 0 < *K*_*u*_ ≤ 0.06 MJ/m^3^ and 0.16 ≤ *K*_*u*_ ≤ 0.21 MJ/m^3^, <*m*_*θ*_> is larger than <*m*_*φ*_>, indicating dominant radial vortex structure. On the other hand, for 0.07 ≤ *K*_*u*_ ≤ 0.14 MJ/m^3^, the circular component of local magnetizations around the core is more dominant, representing a circular vortex structure (see Supplementary Material S1 for details). This result is similar to those found for a 2D planar disk^37^ where a circular vortex structure becomes a radial vortex as the interfacial DMI increases. The radial vortex state found in the hemispherical shells of non-intrinsic DMI suggests that such a curved geometry gives rise to effective DM-like interaction.

In the transient state from vortex to skyrmion (the two cases of *K*_*u*_ = 0.215, 0.22 MJ/m^3^, as shown in Fig. 2a), the local magnetizations at the edge boundary are tilted from the in-plane to the perpendicular orientation with respect to the surface plane. In the skyrmion state (0.23 ≤ *K*_*u*_ ≤ 0.27 MJ/m^3^), the out-of-plane magnetizations are favorable in most areas, i.e., upward at the core and downward in the background. In this state, <*m*_*θ*_> is larger than <*m*_*φ*_>, indicating the existence of the Néel-type domain walls between the upward and downward local magnetizations. In the Néel-type domain walls, in-plane magnetization points towards the skyrmion core, suggesting that the curved-shell system allows for the curvature induced DM-like interaction of a positive DMI constant (see Supplementary Material S2 for details). Also, the size of the skyrmion core decreases with increasing *K*_*u*_. As the *K*_*u*_ increases, the skyrmion width (the length of the domain wall) decreases, which makes the neighboring spins further non-collinear, thus making for higher exchange energy (see Fig. 2d, and Supplementary Material S2 for details). In the perpendicular magnetization state (*K*_*u*_ ≥ 0.28 MJ/m^3^), the higher *K*_*u*_ values favor more perpendicular magnetizations at every local surface in the hemispherical shells, overcoming the in-plane dipolar interaction. Thus, when *K*_*u*_ exceeds 0.28 MJ/m^3^, the skyrmion state totally disappears.

### Phase diagram of spin textures

**Figure 3:**
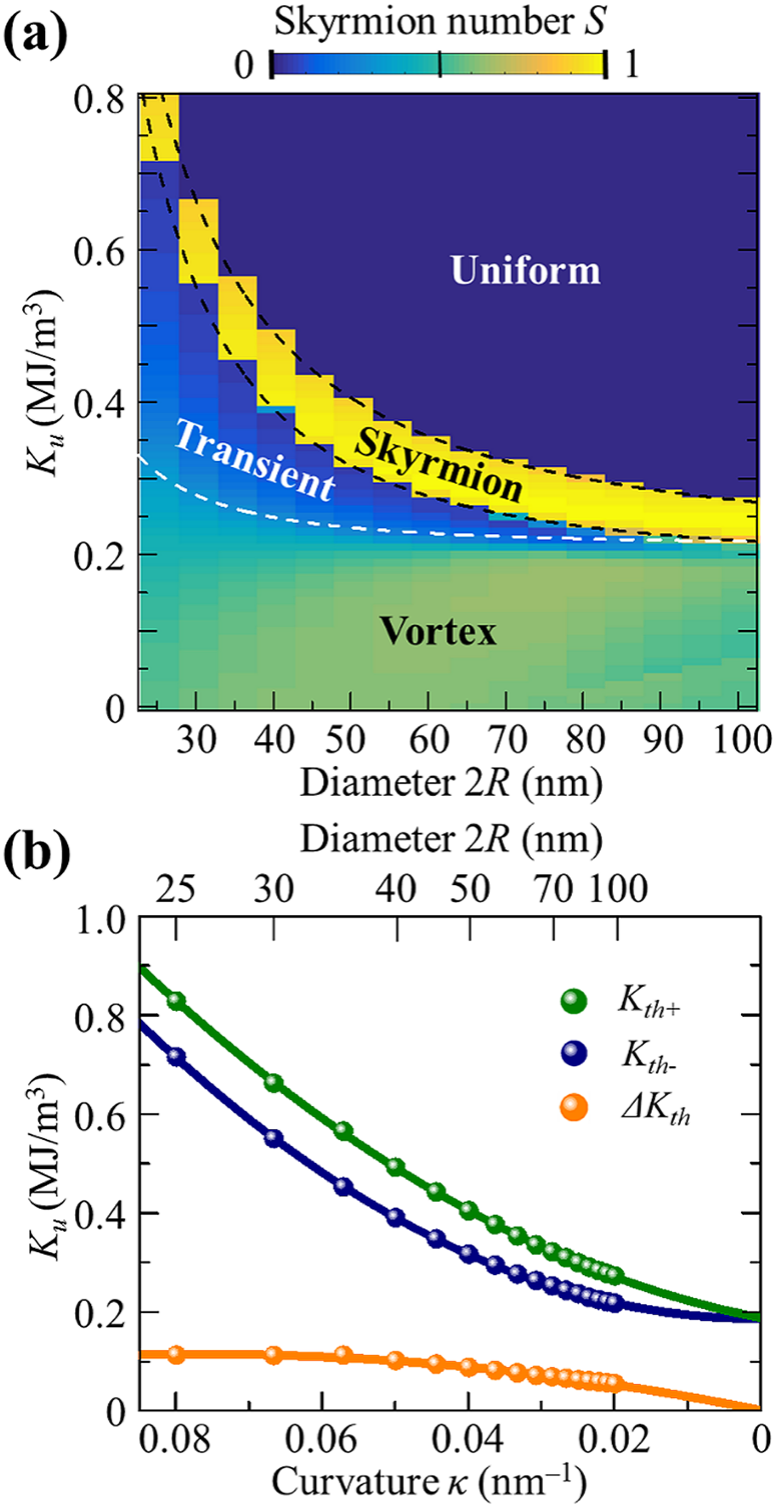
Phase diagram of spin textures. (**a**) Phase diagram with topological charge of meta-stable state as function of shell diameter 2*R* and anisotropy *K*_*u*_. The possible meta-stable states are (1) skyrmion, (2) uniform, and (3) vortex. The black dotted lines represent the upper and lower limits within which skyrmions are well formed, and the white dotted line represents the boundary between the vortex and transient states. (**b**) Upper- and lower-limit *K*_*th*_ values as function of curvature. The solid lines represent the fittings to the data.

Next, in order to elucidate the curvature effect on the formation of skyrmions in hemispherical shells without intrinsic DMI, we constructed a phase diagram of the spin textures by calculating *S* versus both *K*_*u*_ and 2*R*, as shown in Fig. 3a. The characteristic features of the phase diagram are very clear: the vortex, skyrmion, and uniform perpendicular magnetization states appeared in the green-, yellow-, and blue-color regions, respectively. The transient state also existed between the vortex and skyrmion states. It has been reported that in curved geometries, the effective anisotropy constant *K*_*eff*_ can be modified by a curvature-induced anisotropy as $K_{eff} \cong K_{u} - \frac{1}{2}\mu_{0} M_{s}^{2} - \frac{{A_{ex} }}{{R^{2} }}$^33^. Thus, in our system, the boundary between the transition and vortex states can be estimated, according to $K_{u} (K_{eff} = 0)$, to be the white dotted line shown in Fig. 3a. With decreasing 2*R*, the boundary line is slightly shifted to higher *K*_*u*_ values owing to the curvature-induced anisotropy^32,33^. Therefore, the vortex state is always formed in a range of *K*_*u*_ below the boundary (white dashed line). The skyrmion and uniformly perpendicular magnetization states appeared separately depending on *K*_*u*_ for the given 2*R* values. It is interesting that skyrmion formation occurs only in a narrow range of *K*_*u*_, i.e., *ΔK*_*u*_. As 2*R* becomes smaller, the *K*_*u*_ value required for skyrmion formation becomes larger with a wider *ΔK*_*u*_ range, while with increasing 2*R*, *K*_*u*_ becomes smaller and *ΔK*_*u*_ becomes narrower, as shown in Fig. 3a. Since PMA favors co-linear magnetizations while intrinsic DMI leads to non-collinear chiral magnetizations, the curvature of hemispherical shells plays the crucial role of curved-induced DM-like interaction in the formation of skyrmions in the DMI-free shell model. As 2*R* becomes smaller, the curvature-induced DM-like interaction becomes more dominant in the formation of skyrmions, even at higher *K*_*u*_ values. In the hemispherical shell structure, the curvature-induced DM-like interaction plays a crucial role in the formation of skyrmions, just as intrinsic DMI does in planar-geometry structures^33^. Therefore, non-zero intrinsic DMI in such a curved system would further increase the stability of skyrmion formation.

In the phase diagram shown in Fig. 3a, there exists a threshold value $K_{th}^{ + }$ ($K_{th}^{ - }$) above (below) which skyrmions no longer are formed in given shell diameters. Figure 3b further shows the 2*R* (curvature)-dependent *K*_th_ for both the upper and lower limits. Since the estimation of *K*_th_ is somewhat affected by the size of meshes used in finite-element simulations^38^, we kept a constant surface node distance of 1 nm at minimum for all of the shell diameters. The polynomial fitting to the numerical data (solid circles) in the phase diagram gives rise to $K_{th}^{ + }$ = 83.757κ^2^ − 0.100κ + 0.187 MJ/m^3^ and $K_{th}^{ - }$ = 64.381κ^2^ + 2.859κ + 0.188 MJ/m^3^, where *κ* (nm^–1^) is the curvature being defined as the reciprocal of radius, 1/*R*. It is worth noting that both $K_{th}^{ + }$ and $K_{th}^{ - }$ increase as the curvature increases, while at *κ* = 0, $K_{th}^{ + }$ and $K_{th}^{ - }$ both reach a single constant value of *K*_*u*_ = 0.1875 (± 0.0012) MJ/m^3^. This means that skyrmions cannot be formed at *κ* = 0 that corresponds to a flat thin film. This also agrees well with the fact that curved-geometric confinements lead to DM-like contribution to the formation of skyrmions (even without intrinsic DMI)^26^ and enhance the stability of skyrmion formation, as reported in one of our previous studies^27^.

### Excited dynamic modes in skyrmion state

**Figure 4:**
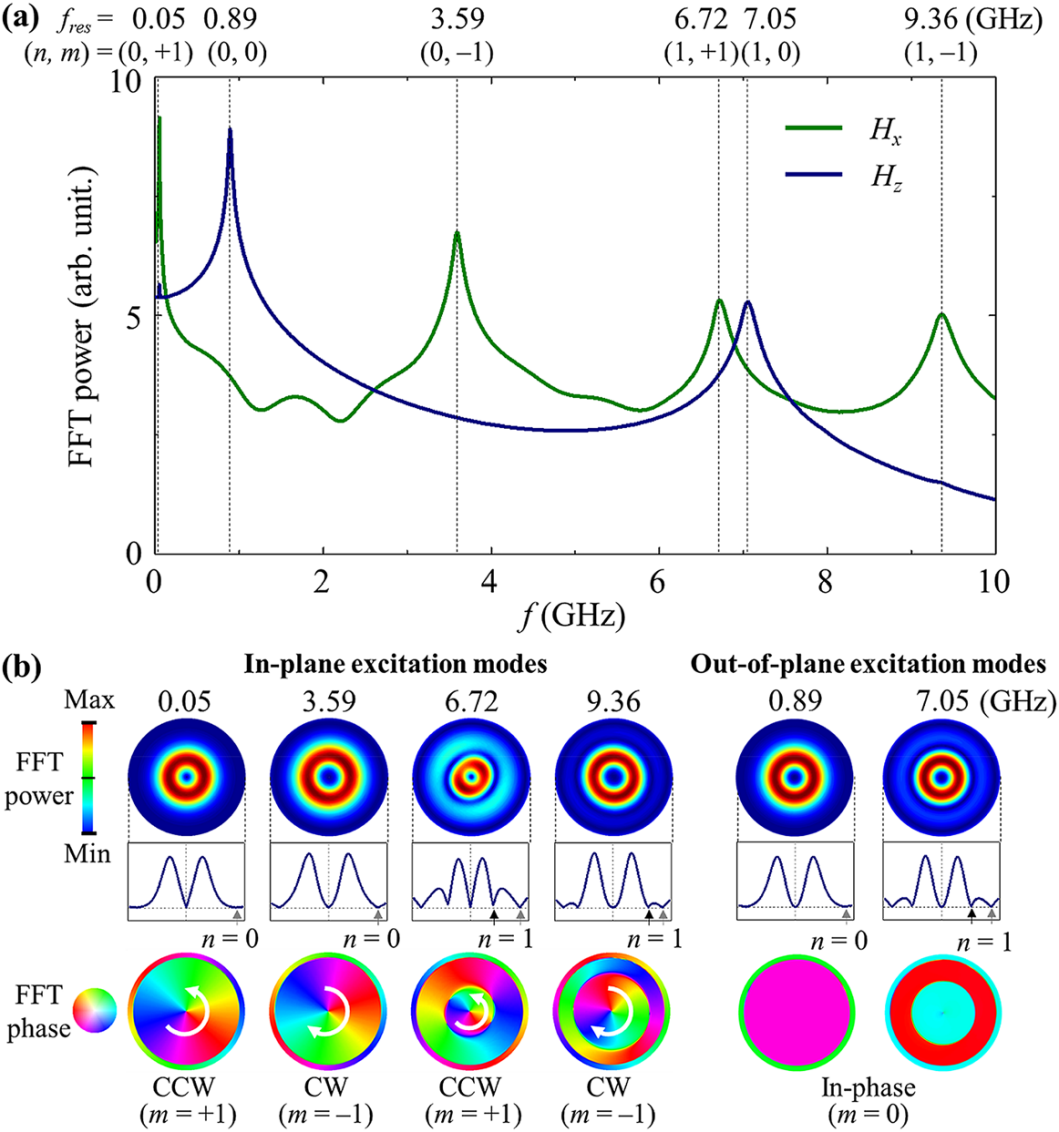
Excited dynamic modes in skyrmion state. (**a**) FFT-power spectra averaged over entire area of hemispherical shell for 2*R* = 100 nm and *K*_*u*_ = 0.25 MJ/m^3^, as obtained from FFTs of temporal oscillations of *m*_*r*_ at all individual nodes, which are excited by each of two different field directions, *H*_*x*_ (green line), and *H*_*z*_ (navy line). (**b**) Unrolled-view images of spatial distributions of FFT powers along with corresponding line profiles across center position (top) and FFT phase (bottom) for in-plane excitation (0.05, 3.59, 6.72, and 9.36 GHz) and out-of-plane excitation (0.89 and 7.05 GHz) modes.

Next, in order to examine the dynamic characteristics of a single skyrmion formed in a hemispherical shell structure, we chose specific values of 2*R* = 100 nm and *K*_*u*_ = 0.25 MJ/m^3^ on the basis of the phase diagram of spin textures (see the 5th state from the left in Fig. 2a). To excite the dynamic modes of the skyrmion, we applied a sinc-function field **H**_sinc_ to the whole shell structure along the *x* (in-plane) and *z* (out-of-plane) axes, where ${\mathbf{H}}_{sinc}(t)={\mathbf{H}}_{0} {\text{sin}}[2 \pi f_{\mathbf{H}}(t - t_{0})]/(2\pi f_{\mathbf{H}}(t - t_{0}))$ with $\left| {{\mathbf{H}}_{0} } \right|$ = 10 Oe, *f*_H_ = 100 GHz, *t*_0_ = 1 ns, and *t* = 100 ns. To obtain a better spectral resolution for all of the excited modes, we used a damping constant of *α* = 0.01. As reported in Ref.^39^, the damping constant can affect the width and amplitude of modes’ resonance spectra, but does not change their resonance frequencies. Figure 4a plots the resultant spectra obtained from the averaged FFTs of the *m*_*r*_ oscillations of all of the individual nodes in the entire volume of a given shell. For the in-plane excitation (green color), there exist four distinct modes at 0.05, 3.59, 6.72, and 9.36 GHz, while for the out-of-plane excitation (blue), there exist only two modes at 0.89 and 7.05 GHz.

To identify all of the excited modes, Fig. 4b displays the spatial distributions of the FFT power and -phase of the local *m*_*r*_ oscillations at the corresponding resonance peaks identified in Fig. 4a. The power line profiles across the skyrmion center along the radial axis clearly inform the position and number of radial nodes *n*, as denoted by the black arrows, while the phase distributions inform the position and number of the azimuthal wave nodes, *m*. We note that one node nearby the shell edge in all of the excited modes (indicated by a gray arrow) was excluded in the numbering of the radial nodes *n*, because the node was caused by the shell edge effect and the curvature-induced DM-like interaction, not by the real eigenmode of the skyrmion. Recently, Mruczkiewicz et al*.*^13^ formulated a classification of the fundamental eigenmodes of the magnetic skyrmions (e.g., gyration, azimuthal, and breathing modes) according to the radial (*n*) and azimuthal (*m*) indices. Accordingly, the excited modes shown in Fig. 4a can be indexed by both *n* and *m*: for the in-plane excitation eigenmodes, the two lower-frequency modes have *n* = 0, and the two higher ones have *n* = 1; each *n* = 0 and 1 has a phase change between − π and π, thus either a CCW or CW rotation sense, and thus is indexed as *m* =  + 1 and *m* = − 1, respectively; or the out-of-plane excitation modes, the radial node number is either *n* = 0 or 1, but with the same azimuthal node of *m* = 0.

**Figure 5:**
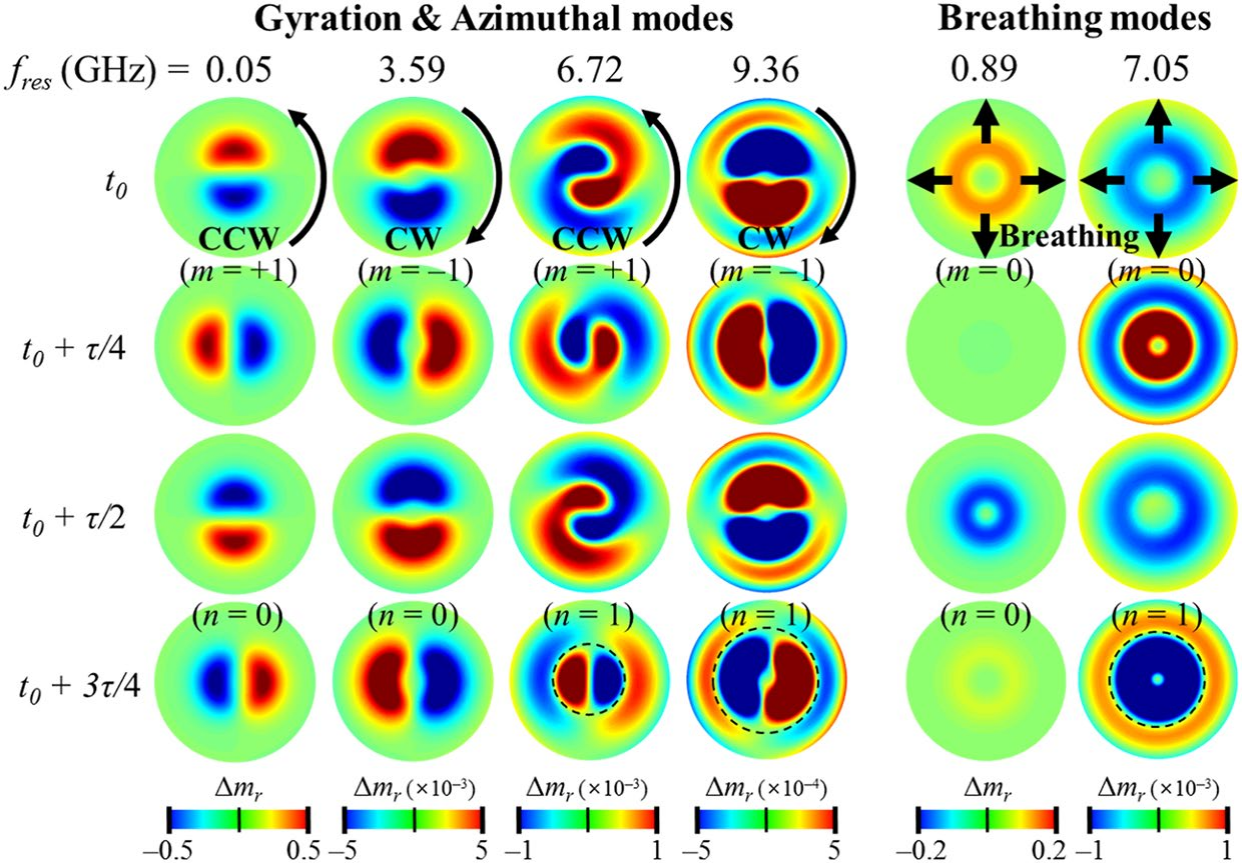
Characteristics of dynamic modes of skyrmion. Snapshot images of *Δm*_***r***_ representative of dynamic eigenmodes of skyrmion: CCW (0.05, 6.72 GHz) and CW (3.59, 9.36 GHz) rotational modes as well as breathing mode (0.89, 7.05 GHz). The first to fourth rows indicate intervals of one-fourth time period.

To clarify the characteristic dynamic modes shown in Fig. 4, we again excited each mode at the corresponding resonance AC field, ${\mathbf{H}}(t) = {\mathbf{H}}_{AC} \sin (2\pi f_{res} t)$, with *f*_*res*_ the resonance frequency. Figure 5 plots the temporal variation of the spatial distributions of *Δm*_*r*_(*t*) = [*m*_*r*_(*t*) − *m*_*r*_(*t* = 0)] in one cycling period *τ*, where *m*_*r*_ (*t* = 0) is the initial *m*_*r*_ state. The characteristic motions of the six modes are clearly manifested in the respective temporal evolutions of their dynamic magnetization profiles. Also, the rotation senses of all of the modes are clearly depicted by the representative snapshot images over one cycle, as shown in Fig. 5. For the in-plane excitation modes, the first (0.05 GHz) and second (3.59 GHz) peaks both have n = 0, and thus the CCW and CW rotational senses are *m* =  + 1 and − 1, respectively. The third (6.72 GHz) and fourth (9.36 GHz) peaks have an additional node in the radial direction (*n* = 1) with the CCW (*m* =  + 1) and CW (*m* = − 1) rotational senses. Although the first and second peaks exhibit similar dynamic behaviors in the change of magnetization *Δm*_*r*_, their dynamic origins are completely different. The FFT power for the 0.05 GHz mode is localized inside the core, while that for the 3.59 GHz mode is spread throughout the background of the skyrmion state (see Supplementary Material S3 for details). Therefore, the 0.05 GHz mode is the gyrotropic mode of the skyrmion core, while the 3.59 GHz mode can be interpreted as an azimuthal spin-wave mode in the outer region of the skymion state^13^. The rotation sense of the gyrotropic motion is CCW for the up-core polarity of the skyrmion state. The rotation sense of the 3.59 GHz mode is opposite to that of the first mode, i.e., CW even with the up-core polarity. In addition, the radius of the core motion at *f*_*res*_ = 0.05 GHz is ~ 8.5 nm, but is as small as ~ 0.077 nm at *f*_*res*_ = 3.59 GHz, which is much smaller than at the 0.05 GHz mode. Thus, the first mode is the gyration mode (*n* = 0, *m* =  + 1) and the second mode is the azimuthal mode (*n* = 0, *m* = − 1), as indexed in Fig. 4a.

The two remaining 6.72 and 9.36 GHz modes are both azimuthal spin-wave modes, with the radial quantization (*n* = 1) corresponding to the CCW (*m* =  + 1) and CW (*m* = − 1) rotation senses, respectively. As can be seen from the boundary separated by the black dashed lines in Fig. 5, the rotation sense of magnetization fluctuation in the inner circle is the same as that in the outer ring region, but with a π phase difference. Here, we note that the core motion’s frequencies coincide exactly with those of the azimuthal spin-wave modes (6.72 and 9.36 GHz). Thus, the rotation senses of the core motion at *f*_*res*_ = 6.72 and 9.36 GHz are not the same as that of the gyrotropic mode, but rather is determined by the rotational sense of the azimuthal modes through their interaction with the given upward core, similarly to the vortex state reported in Ref.^40^. It has been reported that such frequency splitting between the CW (*m* < 0) and CCW (*m* > 0) azimuthal spin-wave modes in skyrmion-state nanodots is caused by DMI-induced spin-wave non-reciprocity^14^.

For the out-of-plane excitation modes, the lower-frequency resonance peak (0.89 GHz) corresponds to the breathing mode^15^ of the skyrmion (*n* = 0, *m* = 0), where the skyrmion radially expands and shrinks with significant fluctuation around its core. The higher-frequency resonance peak (7.05 GHz) with weaker intensity can be viewed as a hybridization mode (*n* = 1, *m* = 0) that combines the breathing mode and the radial spin-wave mode^15^, where the additional node appears along the radial direction. The dynamic magnetization is oppositely oscillating between the core area and the shell edges, with π phase difference. The gyration/breathing mode observed in the sub-GHz region is a unique mode hitherto unseen in the uniform ferromagnetic state (see Supplementary Material S4). All of these modes represent a low-frequency (sub-GHz range) gyrotropic and breathing mode as well as high-frequency radially/azimuthally symmetric spin waves.

## Discussion

Compared with 2D planar geometry, the hemispherical shell structure used in this study has an advantage in terms of choosing materials, because the present geometry structure allows for the formation of stable skyrmions without an intrinsic DMI. With the advance in the development of nanofabrication technologies, such half-spherical caps can be synthesized by depositing a few layered or multilayered films onto 2D arrays of curved-surface dot templates (e.g., spherical polystyrene particles^41,42^ and nanoporous alumina membranes^43^). Perpendicular magnetic anisotropy in such films can also be realized using Co/Pt, Co/Ni, and Co/Pt interfaces. Thus, curvature-stabilized skyrmions can be realized in practical samples. Furthermore, magnetic imaging microscopy techniques such as Lorentz transmission electron microscopy, spin-polarized scanning tunneling microscopy, and high-resolution scanning transmission X-ray microscopy have been used to observe the real-space profiles of skyrmions^9^. Other electrical reading methods were also used to read and detect skyrmions in device applications via magnetic tunnel junction or skyrmion-induced Hall voltages^9^. In addition, it was reported that localized magnetic fields from a magnetic tip allow for direct writing and reading of isolated skyrmions^43^. With the help of advances in the development of 3D nanostructures^41–43^ as well as high-frequency measurements^44^, thereby, the present results offer an alternative opportunity to design spintronic devices based on curved-geometry-induced skyrmion formation. Additionally, since skyrmions possess the eigenfrequencies of dynamic modes in the GHz range, they show promise for magnonics applications such as skyrmion-based tunable resonators^45^.

In summary, we explored curvature-induced DM-like interaction for stabilization of skyrmions in magnetic hemispherical shells and their intrinsic dynamic modes’ excitation driven by AC-oscillating magnetic fields. A phase diagram showed the continuous transitions between three topological magnetization states (i.e., uniform ferromagnetic state, vortex, and skyrmion) according to different perpendicular anisotropy constants and shell curvatures. We made a classification of skyrmion eigenmodes into low-frequency gyrotropic modes and high-frequency spin-wave modes based on the mode symmetry and the number of nodes of the dynamical magnetization profiles in the radial and azimuthal directions. The skyrmion stability condition obtained from the phase diagram can serve as a benchmark for calculations of magnetic skyrmion dynamics in curved geometry as well as a guide to experimentalists for preparation of samples in the magnetic skyrmion ground state.

## Methods

### Micromagnetic simulations

We used the ‘magnum.fe’ code^46^ that utilizes the Landau–Lifshitz–Gilbert (LLG) governing equation^47,48^ as expressed by $\partial_{t} {\mathbf{M}} = - \gamma ({\mathbf{M}} \times {\mathbf{H}}_{{{\text{eff}}}} ) + \alpha ({\mathbf{M}} \times \partial_{t} {\mathbf{M}})$ with phenomenological damping constant $\alpha$, gyromagnetic ratio $\gamma$, and effective field ${\mathbf{H}}_{{{\text{eff}}}}$ to calculate the dynamic motions of individual magnetizations in a curved model system. The energy terms we used in this calculation included the demagnetization (magnetostatic), magnetocrystalline anisotropy, exchange, and Zeeman energy terms, but without intrinsic DMI. The materials used for this model were assumed to be Co interfaced with Pt. The uniaxial PMA was set along the radial axis of all of the local surfaces of shells. The material parameters for Co/Pt were as follows^20^: saturation magnetization *M*_*s*_ = 580 kA/m, exchange stiffness *A*_*ex*_ = 15 pJ/m, damping constant *α* = 0.3, zero DMI constant *D*_*int*_ = 0 mJ/m^3^. The uniaxial PMA constant was varied in steps of 0.01 MJ/m^3^ within the *K*_*u*_ = 0.0–0.8 MJ/m^3^ range.

## Supplementary Information

Supplementary Information 1.

Supplementary Video 1.
