# Supplementary material for: Intrinsic DMI-free skyrmion formation and robust dynamic behaviors in magnetic hemispherical shells
Source: Sci Rep. 2021 Feb 16;11:3886. doi: 10.1038/s41598-021-81624-7 (PMC7887229; doi:10.1038/s41598-021-81624-7)
Supplement: Supplementary file 1 — Supplementary Information 1. [file 41598_2021_81624_MOESM1_ESM.docx]

SUPPLEMENTARY MATERIALS

Intrinsic DMI-free skyrmion formation and robust dynamic behaviors in magnetic hemispherical shells

Jaehak Yang^1^, Claas Abert^2,3^, Dieter Suess^2,3^, and Sang-Koog Kim^1 a)^

*^1^National Creative Research Initiative Center for Spin Dynamics and Spin-Wave Devices, Nanospinics Laboratory, Research Institute of Advanced Materials, Department of Materials Science and Engineering, Seoul National University, Seoul 151-744, South Korea*

*^2^Faculty of Physics, University of Vienna, Austria*

*^3^University of Vienna Research Platform MMM Mathematics - Magnetism - Materials, University of Vienna, Austria*

^a)^ Correspondence and requests for materials should be addressed to S.-K.K. ([sangkoog@snu.ac.kr](mailto:sangkoog@snu.ac.kr)).

**Supplemental Movie (SM)**: Animation of *Δm*_r_ representative of dynamic eigenmodes of skyrmion: CCW (0.05, 6.72 GHz) and CW (3.59, 9.36 GHz) rotational modes as well as breathing mode (0.89, 7.05 GHz).

**S1. Contrasting vortex states according to uniaxial perpendicular magnetic anisotropy constant *K_u_***

In the vortex-state region, at *K_u_* = 0.07 MJ/m^3^, in-plane curling magnetizations around the out-of-plane core magnetization represent a circular vortex structure, whereas at *K_u_* = 0.19 MJ/m^3^, the in-plane magnetizations point in the radial direction toward the core, indicating a radial vortex structure. In addition, there exists an intermediate state between the circular and radial vortex structures at *K_u_* = 0.15 MJ/m^3^. Therefore, the circular and radial vortex states can be characterized by the circular and radial configurations of the local in-plane magnetizations, respectively.


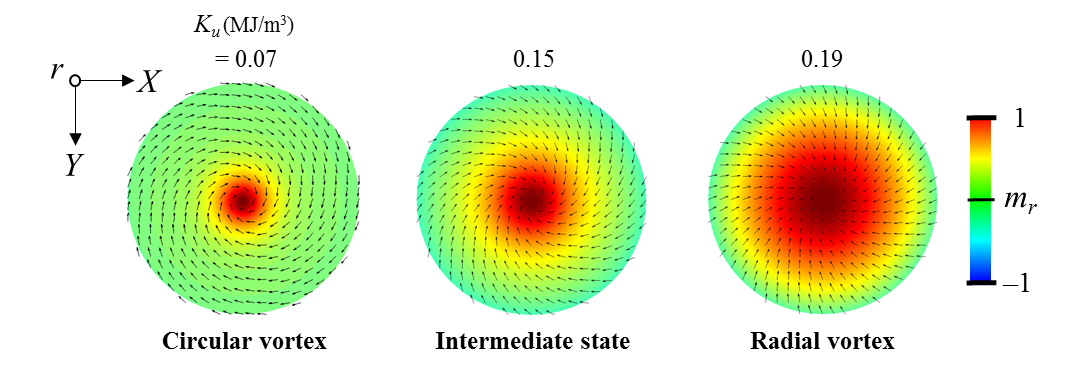


Fig. S1. Unrolled-view images of spatial distributions of radial *m_r_* component of local magnetizations in hemispherical shell of 2*R* = 100 nm for different *K_u_* values as indicated

**S2. Contrasting skyrmion states according to *K_u_***

The variation of the details of the skyrmion structure in the range of *K_u_* = 0.23 – 0.27 MJ/m^3^ is displayed by the three different components of local magnetizations as shown in Fig. S2(a). The azimuthal component *m_φ_* is negligible in the entire range of *K_u_*, but the area of the negative polar component *m_θ_* (blue-color area) around the core region decreases (shrinks) along with the core region with increasing *K_u_*, while the area of the up-core region (red color) shrinks with *K_u_*. The skyrmion diameter *d_sky_* is defined as the distance between two points where *m_r_* = 0 and the width *w_sky_* is defined as the distance between two points where *m_r_* = ±0.5 [see the inset of Fig. S2(a)]. The diameter *d_sky_* and the width *w_sky_* of the skyrmion structure both decrease with *K_u_*. The evolution of the skyrmion structure according to *K_u_* revealed that the width of the Néel-type domain wall and the core size of the skyrmion continuously decrease with *K_u_*.


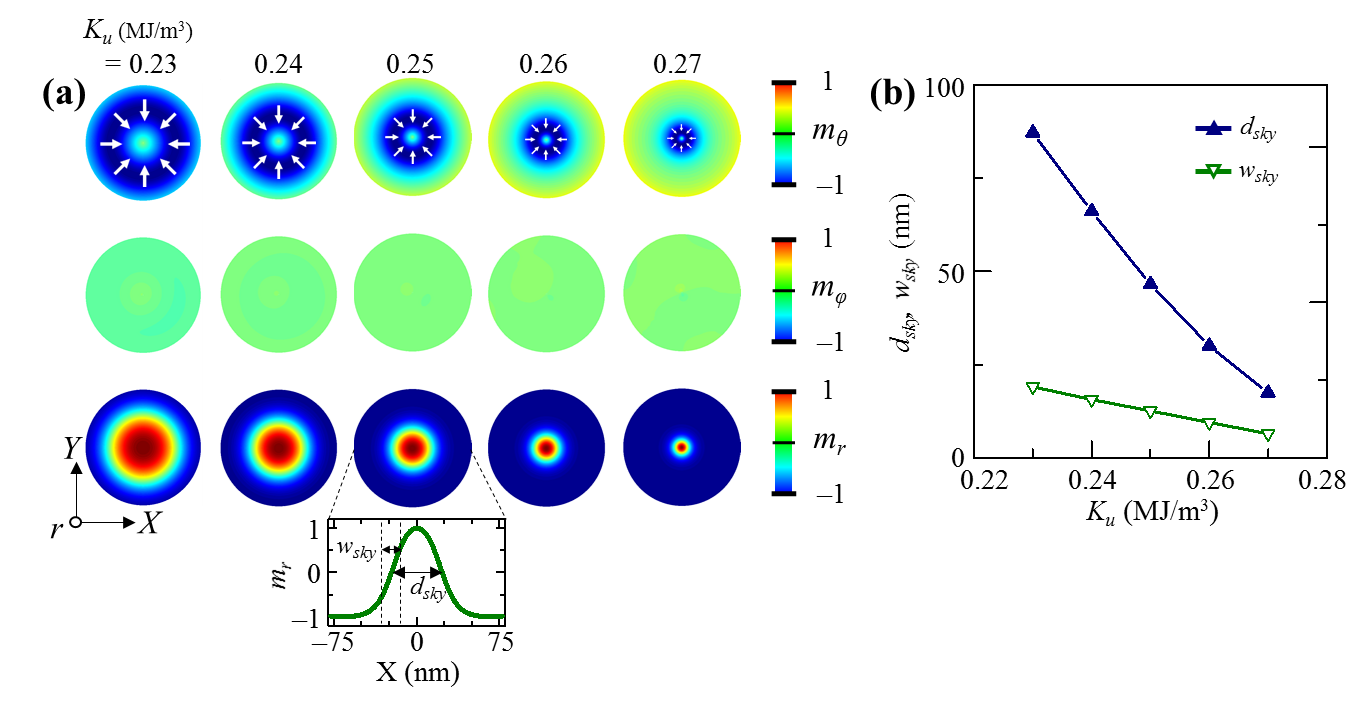


**Fig. S2.** (a) Unrolled-view images of spatial distributions of polar *m_θ_* (top), azimuthal *m_φ_* (middle), and radial *m_r_* (bottom) components of local magnetizations for single skyrmion formed in hemispherical shell of 2*R* = 100 nm for indicated different *K_u_* values. (b) Plots of diameter *d_sky_* and width *w_sky_* of skyrmion as defined in inset of (a), as a function of *K_u_*

S3. Dynamic modes of skyrmion

As shown in Fig. 4(b) of the manuscript, the in-plane excitation modes at 0.05 and 3.59 GHz show very similar distributions of FFT powers for the normal magnetization *m_r_* components, but show opposite rotation senses, CCW and CW, respectively. To clarify the different dynamic behaviors of the two modes (0.05 vs. 3.59 GHz) excited by in-plane AC fields, as shown Fig. 4(b) of the manuscript, we additionally displayed the spatial distributions of the FFT power of the local *m_θ_,* and *m_φ_* oscillations (in-plane magnetization components) at the corresponding resonance peaks of the in-plane excitation eigenmodes, as shown in Fig. S3. As can be found in the FFT-power distribution of the in-plane magnetizations (*m_θ_,* and *m_φ_*), the FFT power of the lowest frequency mode (0.05 GHz) is localized inside the core [see the red square area], while the FFT power of the second lower-frequency mode (3.59 GHz) is spread throughout the background of the skyrmion [see the blue square area]. Therefore, the first lower-frequency mode (0.05 GHz) is a precession of the skyrmion core around its equilibrium position in the dot center (CCW oscillations for the given soliton core polarization *p* = +1). On the other hand, the second lower-frequency rotational mode (3.59 GHz) can be interpreted as an azimuthal spin-wave mode over the background around the skyrmion’s core [SR1]. Since the core motion is induced by its coupling with the excited azimuthal mode (3.59 GHz), the rotation sense is the same as that of the azimuthal mode, i.e., opposite to that of the gyrotropic mode (0.05 GHz).


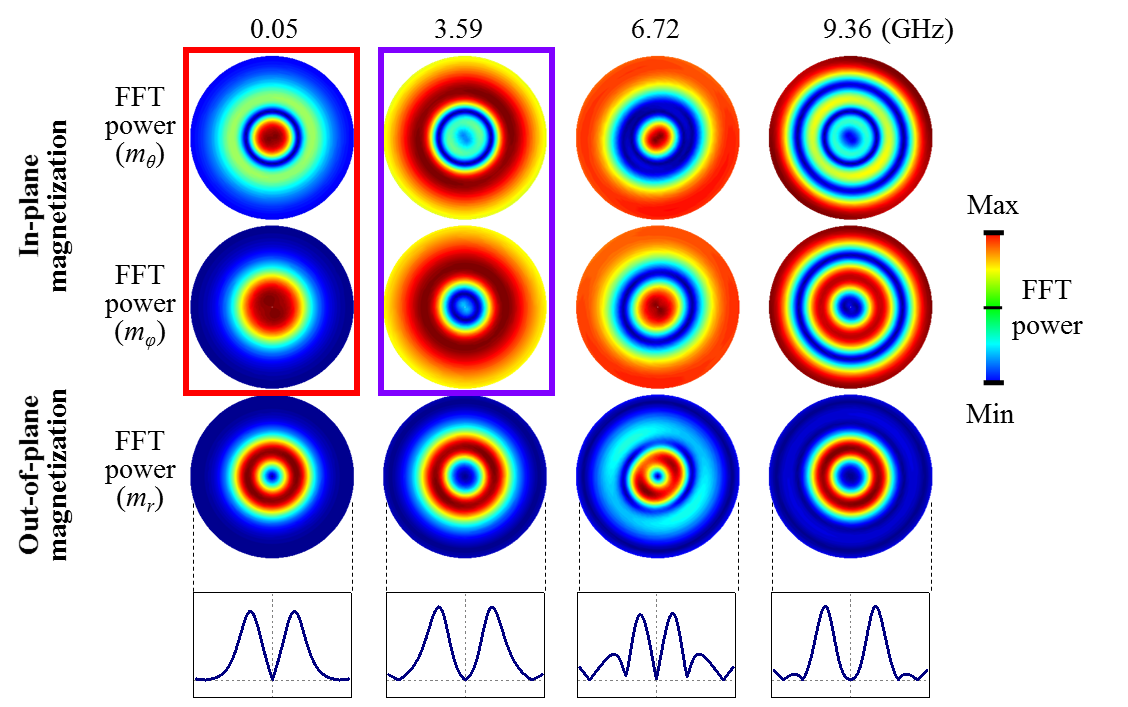


Fig. S3. Unrolled-view images of spatial distributions of FFT power as obtained from FFTs of temporal oscillations of radial *m_r_* (first row), polar *m_θ_* (second row), and azimuthal *m_φ_* (third row) components of local magnetizations excited by in-plane AC fields of corresponding resonance frequencies of 0.05, 3.59, 6.72, and 9.36 GHz. The last row corresponds to the line profile of the FFT power (*m_r_* component) across the core center.

**S4. Spin-wave modes of uniform magnetization state.**

For perpendicularly uniform magnetization states (*K_u_* = 0.274 ~ 0.8 MJ/m^3^), we also excited spin-wave modes by the same sinc-function field **H**_sinc_ to the whole-shell structure along the *x* (in-plane) and *z* (out-of-plane) axis. Figure S4 shows frequency spectra representing the excited two modes at 8.73 and 9.45 GHz. The in-plane excitation mode (8.73 GHz) is the FMR mode in which the center part is strongly excited, and the out-of-plane excitation mode (9.45 GHz) is the breathing mode localized to the edge [See Fig. S4(b)].


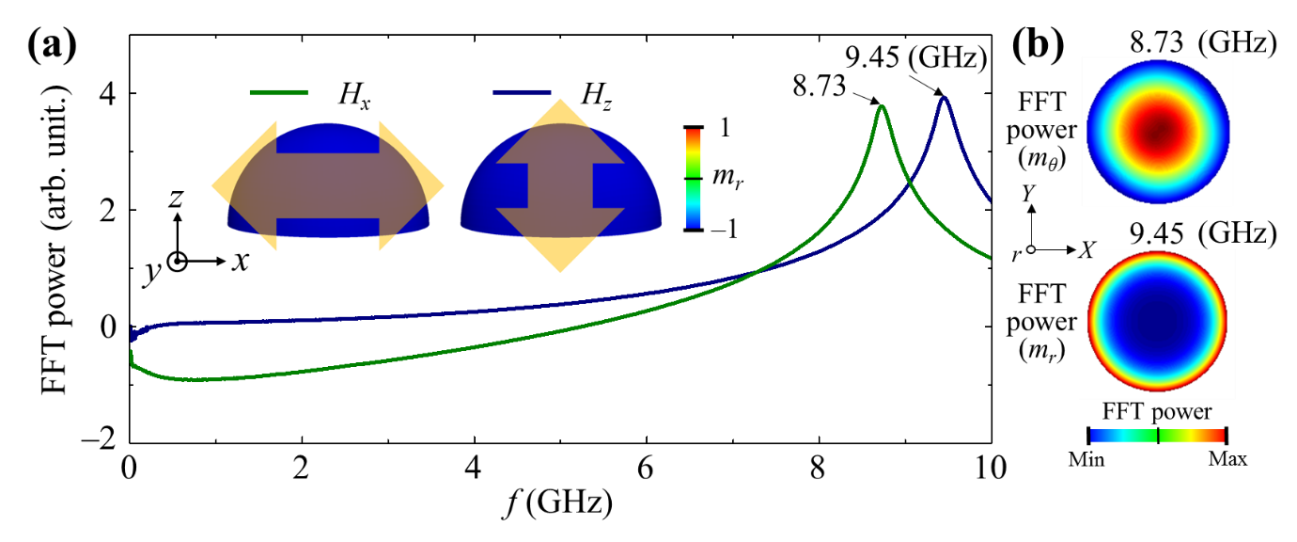


**Fig. S4.** (a) Frequency spectra of FFT power as obtained from FFTs of temporal oscillations of *m_r_* at all individual nodes excited by sinc field applied along either *x-* or *z*-axis. (b) Unrolled-view images of spatial distributions of FFT powers for 8.73 GHz and 9.45 GHz excited modes.

Supplementary References

[SR1] Mruczkiewicz, M. *et al*. Spin excitation spectrum in a magnetic nanodot with continuous transitions between the vortex, Bloch-type skyrmion, and Néel-type skyrmion states. *Phys. Rev. B* **95**, 094414 (2017).
